# Factors Affecting Milk Productivity, Milk Quality and Dairy Cow Health

**DOI:** 10.3390/ani14243707

**Published:** 2024-12-23

**Authors:** Michał Bednarski, Robert Kupczyński

**Affiliations:** 1 Department of Epizootiology and Clinic of Bird and Exotic Animals, Faculty of Veterinary Medicine, Wroclaw University of Environmental and Life Sciences, 47 Grunwaldzki Sq., 50-366 Wroclaw, Poland; 2 Department of Environment Hygiene and Animal Welfare, Faculty of Biology and Animal Science, Wroclaw University of Environmental and Life Sciences, 38c Chelmonskiego St., 50-375 Wroclaw, Poland

Milk and dairy products are considered important sources of nutrients in human nutrition due to their content of high-quality protein, minerals, vitamins, and energy [1,2]. The growing demand for dairy products necessitates an increase in the average milk yield per cow. The composition of milk is determined by various factors, including genetics (breed), physiology (age, stage of lactation, health status), and environmental conditions (nutrition, season, housing conditions) (Figure 1). An increase in milk yield can be achieved through genetic selection, as well as improvements in cow nutrition and management. Nutritional modifications in the composition and proportions of individual components can relatively quickly lead to an increase in milk yield and changes in its composition. One of the biggest issues affecting milk yield is poor udder health, particularly due to mastitis [3]. Better mastitis management can help improve milk quality and promote more responsible use of critical antimicrobial agents on dairy farms.

Milk fat has a complex composition of fatty acids (FAs) with varying numbers of carbon atoms and different degrees of unsaturation. Supplementing cows’ diets with lipids offers benefits such as improved energy balance and influences milk composition. By using specific feeds, it is possible to modify the fatty acid profile of cows’ diets towards producing functional foods. Fatty acids (FAs) are a key component of milk fat. Increasing the content of n-3 FAs in milk, especially α-linolenic acid (ALA, 18:3n-3), eicosapentaenoic acid (EPA, 20:5n-3), and docosahexaenoic acid (DHA, 22:6n-3), could provide significant health benefits for humans. In general, milk and dairy products are major sources of both healthy and unhealthy FAs in human nutrition. Conjugated linoleic acid (CLA) is a group of polyunsaturated fatty acids found in beef, lamb, and dairy products, existing as positional and stereo-isomers of octadecadienoate (18:2). *cis*-9, *trans*-11 CLA and *trans*-10, *cis*-12 CLA are considered the most abundant and essential isomers associated with health benefits. In the review by Wang et al. [4,5], the authors focused on the unique properties of CLA, particularly its anti-cancer, anti-inflammatory, immune-regulating, and lipid metabolism-regulating effects, which may potentially contribute to the growth and health of infants.

The sources of fatty acids in ruminant milk fat are the fatty acids produced during ruminal processes, lipids from the diet, and mobilized body fat reserves. Supplementing the diet with lipid sources rich in polyunsaturated fatty acids (PUFAs), such as oilseeds and/or vegetable oils, fish oil, and microalgae, as well as altering the concentrate-to-forage ratio and allowing cows to graze on pastures, can increase the content of CLA in milk and dairy products [5,6]. Using statistical methods, accurate and precise predictions of milk fat production and proportion, and FA profiles, can be achieved by considering cows’ production and dietary characteristics [6]. The first study to quantitatively examine the effects of lipid-supplemented diets, characterized by their FA profile, on milk fat production and proportion was presented in this research [6]. While supplementation with natural additives may improve milk yield and composition, the results are not always conclusive [7,8]. The primary benefit may be an improvement in the milk fatty acid profile and blood plasma antioxidant status [8].

The content of odd- and branched-chain fatty acids (OBCFAs) in milk is considered from both a health perspective and as an indicator of ruminal metabolism [9]. The presence of OBCFAs in cow milk fat largely results from bacterial processes in the rumen, although endogenous synthesis and/or the conversion of certain fatty acids due to lipomobilization also play a role. The concentration of OBCFAs in cow milk fat depends on various factors, including the feed ration composition (the proportion of roughage to concentrate), rumen pH, and ammonia concentration [9]. More research is needed to address discrepancies in OBCFA content and their correlations with various factors to optimize approaches to increasing the OBCFA content in milk and dairy products, thus improving animal and human health. Analyzing the OBCFA profile is a promising, non-invasive method for assessing ruminal fermentation processes. Certain OBCFAs in milk fat could be markers for rumen acidosis and microbial protein flow into the duodenum. Additionally, OBCFAs are among the fatty acids associated with body fat mobilization. However, further research is required to better understand the transfer of OBCFAs from the rumen to milk, which could improve the accuracy of predicting rumen changes or preventing metabolic disorders. Moreover, analyzing essential oils and OBCFAs in ruminant milk could assist in determining methane (CH_4_) emissions [9,10]. Natural compounds added to cow diets may reduce methane emissions into the environment, offering a viable nutritional solution for enhancing sustainable milk production.

One of the most significant metabolic disorders in dairy cows is subclinical ketosis. This condition leads to substantial economic losses and increases the risk of other diseases [11]. Ketosis occurs in two forms, Type I and Type II, which are distinguished not only by their pathogenesis but also by the time of onset. In a study by Chisato et al. [12], the occurrence of ketosis was analyzed in Postpartum Subclinical Ketosis in Dairy Herds in Hokkaido, Japan. Their analysis showed that the overall incidence of subclinical ketosis in cows was 17.6%. Type II ketosis, classified in the study based on its occurrence within the first 14 days postpartum, had an incidence rate of 20.2%, while Type I ketosis was had a lower incidence rate of 16.5%. The occurrence of this condition was linked to the management system, feeding practices, and lactation stage.

Mastitis is another important group of diseases affecting both the quality and quantity of milk. Currently, regulations in many countries aim to limit the use of antibiotics, which, in the case of cows, will particularly impact dry cow therapy (DCT). Such measures enforce stronger emphasis on prevention and good farming practices, including those related to milking [13]. Therefore, research on risk factors allows for a better understanding of how to reduce the risk of mastitis. Two studies presented in this Special Issue address this topic. One of these studies, conducted by Cortinhas et al. [14], revealed a strong positive relationship between the somatic cell count in bulk tank milk and dairy farmers’ knowledge about milk quality and mastitis control and their socioeconomic characteristics. The type of milking equipment and hygiene practices also proved to be significant factors.

International organizations have expressed concerns about the excessive use of antimicrobial agents and their impact on antimicrobial resistance, which affects both human and animal health. An important aspect of animal health is its influence on the need for antibiotics. In the publication by Kupczyński et al. [15], the results of monitoring studies on antibiotic usage on medium and large dairy cattle farms, as well as in beef cattle herds, are presented, broken down by the type of therapy (general therapy, LC therapy, and DC therapy) and the type of antibiotic used. The studies indicate that the largest amount of antibiotics is used on large dairy farms, followed by medium-sized farms, with the least usage in beef cattle herds. On large dairy farms, both relatively and absolutely, the highest amounts of critical antibiotics were also used, including for drying off cows. Selective dry cow therapy (SDCT) allows for a reduction in preventive antimicrobial use during the dry period [16].

We hope that the papers collected in this Special Issue will help guide future projects and spark discussions on the topics covered. Dairy production systems must continue to ensure good performance and profitability while also reducing their environmental impact and maintaining high standards of animal health and welfare. In the future, efforts must continuously be made to address issues related to animal welfare and improve environmental, livestock, and human health under the One Health approach.

## Figures and Tables

**Figure 1:**
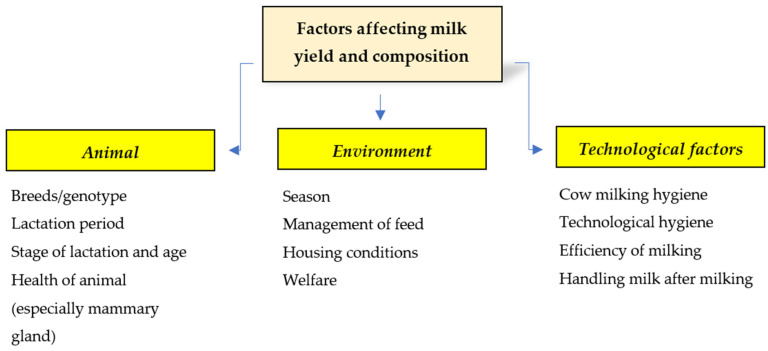
Impact of key factors on cow milk yield and composition.
